# The Effect of Modified Biolasol Solution on the Efficacy of Storing Isolated Porcine Kidneys

**DOI:** 10.1155/2018/7465435

**Published:** 2018-11-12

**Authors:** Aneta Ostróżka-Cieślik, Barbara Dolińska, Florian Ryszka

**Affiliations:** ^1^Department of Pharmaceutical Technology, School of Pharmacy and the Division of Laboratory Medicine in Sosnowiec, Medical University of Silesia, Katowice, Poland; ^2^“Biochefa” Pharmaceutical Research and Production Plant, Sosnowiec, Poland

## Abstract

Biolasol is a newly developed solution for storing the liver, pancreas, kidneys, and heart by simple hypothermia. It exhibits high efficacy in maintaining structural and functional integrity of the graft prior to its transplantation. The solution was modified by the addition of ascorbic acid (0.088g/l) and ascorbic acid with prolactin (1 *μ*g/l PRL + 0.088g/l vitamin C). The effectiveness of the obtained solutions in the protection of nephrons of isolated porcine kidneys was assessed based on the analysis of the activity of ALT (alanine aminotransferase), AST (aspartate aminotransferase), and LDH (lactate dehydrogenase) as well as lactate concentration determined in perfundates collected after 2 h (0′ and 30′ preservation) and 48 h (0′ and 30′ preservation) of graft storage. It has been found that the synergistic action of Biolasol components determines the integrity and stability of cell membranes, which in turn affects the proper functioning of the organ after transplantation. The addition of ascorbic acid and prolactin to Biolasol affects the maintenance of the normal cytoskeleton of the stored graft.

## 1. Introduction

Biolasol is a newly developed solution for storing the liver, pancreas, kidneys, and heart by simple hypothermia. It exhibits high efficacy in maintaining structural and functional integrity of the graft prior to its transplantation. Biolasol is an extracellular fluid with a sodium concentration of 105 mmol/l and potassium concentration of 10 mmol/l. Dextran70 (colloid osmotic) affects the maintenance of the correct volume of fluids in the intravascular space. Disodium edetate (EDTA) complexes multivalent metal cations. By chelating Ca^2+^ ions, it blocks the activation of zymogens involved in the coagulation process. In complex with Fe^2+^ ions, it reduces the risk of damage caused by the activity of the hydroxyl radical formed in the presence of iron with H_2_O_2_ in the Fenton reaction. Iron chelators reduce the release of lipid peroxidation products, which minimizes the inflammatory response and the influx of neutrophils into the graft. Magnesium fumarate minimizes cell damage during ischemia and reperfusion. Sodium bicarbonate functions as a buffer system and helps maintain the proper acid-base balance. Glucose is involved in the renewal of ATP [1–4].  Table 1 compares the composition of Biolasol with other fluids available on the world market [5].

Biolasol limits the effects of organ ischaemia and prevents its dysfunctions resulting from rapid cooling. Oxygen deficiency and the switch of cells to anaerobic metabolism reduce ATP reserves and impair the sodium-potassium pump. There occurs an uncontrolled inflow of sodium and calcium to the cell. Ca^2+^-dependent proteases and phospholipases are activated causing lysis of the cell membrane and damage to ion channels. A decrease in pH, lactate accumulation, and inhibition of oxidative phosphorylation are also observed. Free oxygen radicals are generated, including the superoxide radical, which is toxic to the lipid membranes of the cell and damages the structure of proteins and enzymes. As a consequence, it can lead to severe damage to organs. Biolasol enables restoring their proper functioning after transplantation [1–6].

A number of clinical trials were carried out to assess the effectiveness of Biolasol, also in relation to commonly used perfusion and organ preservation fluids. Its effectiveness was not worse than that of HTK, UW, and Viaspan fluids. Biolasol protects grafts against ischemic damage in a similar way as the aforementioned solutions. It has been found that Biolasol provides better homeostasis of isolated porcine kidneys during storage compared to the HTK solution [2]. Jóźwik et al. [7] transplanted into patients 42 kidneys which had previously been rinsed and stored in Biolasol and UW solutions. They demonstrated comparable effectiveness of both fluids [7]. Cierpka et al. performed comparative studies of the effectiveness of Biolasol and Viaspan in the procedure of kidney autotransplantation in 12 pigs. They have shown that the used solutions protect the kidneys from ischemia-reperfusion injury in a similar way [3]. Based on our histopathological examinations, we have found that adding prolactin to the HTK preservation fluid minimizes hepatocyte damage in the model using an isolated rabbit liver [8]. Cierpka et al. confirmed by means of histopathological examination that the structure of the isolated porcine kidney cortex was not damaged after using Biolasol [1].

Biolasol was modified by the addition of ascorbic acid and ascorbic acid with prolactin. Prolactin, a hormone secreted by pituitary cells, and an exogenous antioxidant, vitamin C, were used in the modification. Ascorbic acid plays an important role in maintaining the appropriate oxidation-reduction potential in cells and neutralizes the reactive forms of oxygen and nitrogen resulting from cellular metabolism. It occurs both outside and inside the cells. The normal concentration of ascorbic acid in the plasma is over 17 *μ*mol/l, usually 45-80 *μ*mol/l, whereas in leukocytes and platelets it is approximately 1480 *μ*mol/l. In turn, prolactin (PRL) is a protein hormone and a strong cytokine with a broad spectrum of biological activities. It acts as an immunoregulator in cell proliferation and differentiation and is an apoptosis inhibiting factor. It enhances the expression of IL-2 receptors on lymphocytes and stimulates the production of antibodies by B-lymphocytes. It affects the production of lysozyme and lowers the high ceruloplasmin level induced by inflammatory reactions [9, 10]. Ryszka et al. administered prolactin subcutaneously at a dose of 25 *μ*g/kg of body weight in rats. They have found that the distribution of prolactin in selected organs and tissues decreases in the following order: milk gland> blood> pituitary> ovaries> lungs> liver> cranial bone> spleen> heart> kidneys> muscular tissue> adenose> adipose tissue> brain [11]. Prolactin acts by means of specific PRLRs, belonging to type I cytokine transmembrane receptors. Specific PRLRs are located at various places in cells and tissues [12]. The presence of PRL receptors was found in the proximal renal tubules and in the nephron, in the thick section of the ascending arm of the Henle loop, and in the distal tubule and the collecting duct [13]. Ibarra et al. have found that PRL is a natriuretic hormone that interacts with the renal dopaminergic system in inhibiting Na+, K+, and ATP-ase in the proximal renal tubules [14]. Prolactin may affect the filtration rate in the renal glomerulus and the renal plasma flow [15]. It has also been found to affect the proliferation of renal tubular epithelium [16]. It is suggested that PRL receptors are also located in the three zones (cytoplasm of cells, zona glomerulosa, and zona fasciculata) of the adrenal cortex [17].

An important consequence of renal ischaemia is the disorder of apoptosis and repair processes within the renal tubules. The loss of integrity of the cytoskeleton of cells, detachment of the brush border of the proximal tubules, and disturbance of expression of adhesion particles are observed. The C3 segment of the proximal tubule of the nephron and the thick ascending limb of Henle's loop are the most sensitive to ischemia. Damaged cells of the tubules peel off and clog the lumen of the tubules, which causes leakage of filtrate into the lumen of the capillary vessels and a decrease in glomerular filtration [18].

The effectiveness of the modified Biolasol fluid in the protection of nephrons from the effects of ischemia and hypoxia was assessed based on the study of aminotransferase activity, LDH activity, and lactate concentration in the perfusates taken from the renal vein. AST and ALT belong to cellular enzymes, whose increased activity correlates with the increased permeability of cell membranes and/or indicates the breakdown of cells. Aspartate aminotransferase is in 30% present in the tissues of the body as a cytoplasmic isoform (AST1) and in 70% as a mitochondrial isoform (AST2). The increase in its activity is mainly related to the damage of mitochondrial membranes. In turn, alanine aminotransferase is produced in the renal tubular epithelium, and its increased activity indicates damage to the cytoplasmic membranes. Lactate dehydrogenase is located in the cytoplasm of the cell, and its activity increases when cell/tissue necrosis occurs [19]. Lactates are produced in the tissues of the whole body in the process of anaerobic glycolysis. The amount of released LDH and lactates indicates the degree of acidification of the intracellular environment. The determined values of the abovementioned markers in perfusate samples may be helpful in determining the extent of kidney damage during storage [20, 21].

The aim of the study was to evaluate the modified Biolasol solution in terms of the protection of nephrons of isolated porcine kidneys based on the analysis of the activity of ALT (alanine aminotransferase), AST (aspartate aminotransferase), and LDH (lactate dehydrogenase) as well as lactate concentration determined in perfundates collected after 2 h and 48 h of graft storage.

## 2. Materials and Methods

**Figure 1:**
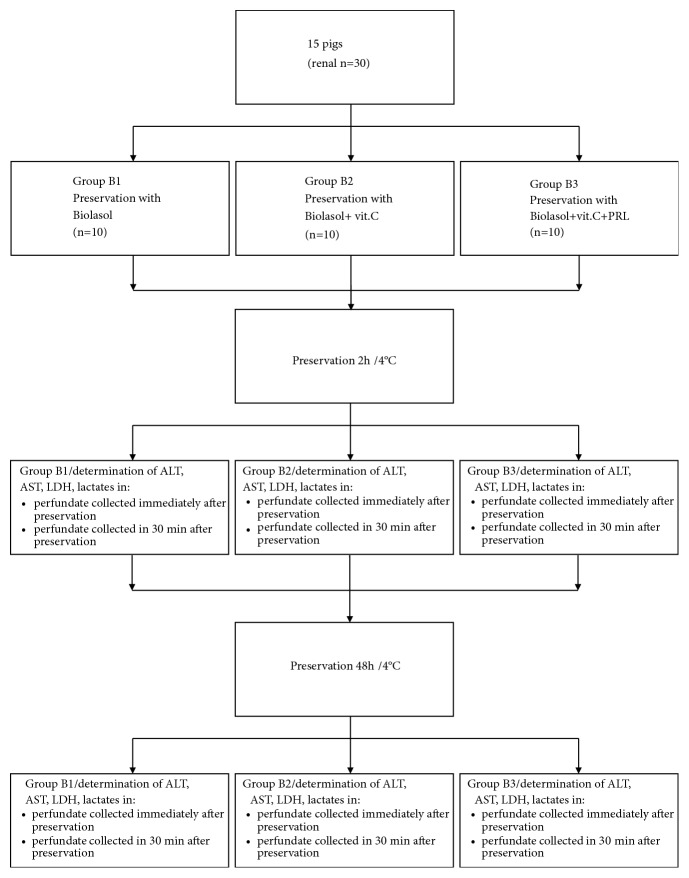
Study design.

The study used Biolasol solution (FZNP “Biochefa”, Poland) and Biolasol modified by the addition of porcine prolactin - 1 *μ*g/l (FZNP “Biochefa”, Poland) and/or ascorbic acid – 0.088g/l (PLIVA Pharmaceutical Company, Cracow, Poland). The study used 30 kidneys from 15 adult Great White Poland pigs weighing 90-110 kg, aged 175-180 days. The kidneys were collected in the slaughterhouse of the Meat Plant H.A.M in Radzionków. After collection, the kidneys were cannulated and stored in a suitable preservation solution (Biolasol, Biolasol+vit.C, or Biolasol+vit.C+PRL) at 4°C for 2 hours (it was the time necessary to transport the organ from the slaughterhouse of H.A.M Meat Plant in Radzionków to the laboratory). The kidneys were then rinsed under the pressure of 73,5 mmHg H_2_O with the following solutions: Biolasol, Biolasol + vit.C, and Biolasol + PRL + vit.C. The perfusate samples were collected from the kidney vein at 0 and 30 minutes of perfusion. After 30 minutes, the kidneys were cooled and placed in a sterile bag filled with 500 ml of appropriate preservation solution (Biolasol, Biolasol+vit.C, or Biolasol+vit.C+PRL) for 48 hours (maximum time of organ storage in Biolasol). After this time, activities related to renal perfusion were repeated. In the perfusate samples, the activity of the released indicator enzymes, namely, aspartate aminotransferase (AST), alanine aminotransferase (ALT), and lactate dehydrogenase (LDH), as well as lactate concentration was determined by spectrophotometric methods using the bioMérieux diagnostic kit, Lyon, France (Figure 1).

The normality of the distribution of variables was checked using the Shapiro-Wilk criteria. Comparison among groups was performed using the Kruskal-Wallis test for nonparametric continuous variables, or variance (ANOVA) for parametric continuous variables. The calculations were made using Statistica version 8.0 software (StatSoft, Poland).

## 3. Results

On the basis of the conducted tests (Table 2), it has been found that the increase in AST activity in the modified solution perfusates is accompanied by a marked lower increase in ALT activity, which translates into the ratio of the activity of these enzymes in porcine serum under physiological conditions (AST - 32-84 U/l, ALT - 31-58 U/l) [2]. It has also been observed that, after 2 hours of storage, both in perfusates of Biolasol modified with the addition of vitamin C and Biolasol modified with the addition of vit.C and PRL, ALT activity remained at the physiological level: 34.7 U/l vs. 43.7 U/l. After 48 h of storage, there was a slight decrease in ALT activity in both cases: 29.6 U/l (~14.7%) vs. 43.2 U/l (~1.1%). The difference is not significant. The obtained results of alanine aminotransferase activity are lower compared to its activity in Biolasol solution perfusates (70.6 U/l-2h, 68.6 U/l-48h). The difference is not significant.

Aspartate aminotransferase (AST) activity remained at the physiological level in the perfusates of all analysed solutions after 48 hours of renal storage. An increase in this parameter was observed after 2 h of storing the graft in Biolasol modified with the addition of vit.C and PRL - 103.4 U/l (~18.8% vs. norm 84 U/l). This may indicate that nephrons were damaged in the early period of kidney storage, presumably as a result of increased expression of prolactin receptors. After 2 hours of 30′ preservation using Biolasol modified with the addition of ascorbic acid and prolactin, the AST activity decreased by 38%. Rinsing the blood off the organ might have resulted in the restoration of intracellular calcium homeostasis and improvement of mitochondrial cell activity [22, 23].

The physiological norm of lactate dehydrogenase activity in porcine serum is 380-634 U/l [2]. After 48 hours of storage, LDH activity determined in the perfusates of all solutions oscillates within the normal range. A slight increase in LDH activity (~12%) was reported after 2 hours of storing the kidneys in Biolasol solution.

Metabolic acidosis is caused by an increase in serum lactate level above 2 mmol/l. In all the analysed perfusate samples, the lactate concentration was within the normal range. The lowest concentration of this parameter was determined in the perfusates of Biolasol modified with the addition of vit.C and PRL at 30 minutes of perfusion after 2 h and 48 h of storage (0.2 mmol/l).

## 4. Discussion

High aminotransferase activity, LDH activity, and an increase in lactate concentration may indicate renal ischemic damage and may correlate with the loss of secretory function after transplantation [2, 21, 24]. A similar relationship has been observed by Li et al. [25]. Hypoxia of renal tubule cells during cold ischaemia results in a significant LDH release [26]. Renal damage also causes the release of AST and ALT located in the proximal tubule [27, 28]. The abovementioned parameters were significantly reduced during preservation in Biolasol, Biolasol + vit.C, and Biolasol + vit.C + PRL. Biolasol solution and its modifications were used in maintaining the structural and functional integrity of kidneys under hypoxic conditions. One of the possible protective mechanisms may be an antioxidant effect.

Prolactin, besides fulfilling many biological functions, has a pleiotropic effect. On the basis of the conducted research, it is supposed that PRL participates in the removal of free oxygen radicals (ROS) generated in the cellular space. At present, the mechanism of its operation in this aspect is unknown [29]. It is suggested that PRL may act as an antioxidant in enhancing endogenous antioxidants [29]. It has been found that prolactin indirectly influences the increase of glutathione (GSH) concentration in the cell by stimulation of, e.g., transcription factors, including those regulating insulin secretion. This increase may result from increased glutamate-cysteine ligase (GCL) activity, which catalyses the key step of GSH synthesis [30, 31]. The results of our research suggest that PRL may also exhibit a synergistic effect with exogenous antioxidants, i.e., vitamin C.

The obtained parameters suggest a positive effect of ascorbic acid on the integrity of the cytoskeleton of the stored graft. During ischemia, the formation of reactive oxygen species (ROS) is activated, which reduces the effectiveness of antioxidative systems. With a large amount of ROS, lipid peroxidation reactions, which are one of the causes of cellular damage, are triggered. Polyunsaturated fatty acids included in phospholipids, which are the building blocks of cell membranes, undergo peroxidation [32]. Supplementing the liquid with an exogenous antioxidant in the form of ascorbic acid supports the weakened graft antioxidant system. Consequently, vitamin C reduces the formation of ROS and has a protective effect on cell integrity. Lloberas et al. carried out research which has shown that the administration of vitamin C during kidney transplantation in a rabbit reduces the concentration of lipids and myeloperoxidase and improves organ function [33]. It has been noted that the administration of vitamin C during kidney transplantation in humans significantly reduces damage caused during reperfusion [34]. In addition, it has been found that preservation solutions modified with vitamin C are more durable [35].

Prolactin may indirectly affect the dilation of renal vessels and, as a consequence, increase the glomerular filtration rate [36]. It is suggested that PRL blocks the inflow of Ca^2+^ to the inside of the cells. Increased calcium concentration in the cell results in the activation of intracellular enzymes that cause the degradation of phospholipids and increase the permeability of the cell membrane.

A number of our studies indicate the hepatoprotective and nephroprotective effects of prolactin [8, 20, 21]. PRL protects the structure and function of cells against the negative effects of ischemia and hypoxia [20, 37]. The addition of this hormone to preservation solutions affects the regeneration of cells after hepatectomy and nephrectomy. It also ensures the integrity of the cell membrane and contributes to the maintenance of normal balance of ions and normal morphological parameters of the liver and kidneys [20, 37]. The addition of 1 *μ*g/l rh-PRL to Biolasol solution reduces ALT and AST activity during reperfusion [20].

## 5. Conclusions

The synergistic action of Biolasol components determines, inter alia, the integrity and stability of cell membranes, which in turn affects the proper functioning of the organ after transplantation. The addition of ascorbic acid and prolactin to Biolasol solution affects the maintenance of the normal cytoskeleton of the stored graft.

## Figures and Tables

**Table 1:** Composition of preservation solutions.

Component	Biolasol	Viaspan	IGL-1	HTK	Celsior
IC/EX	EX	IC	EX	EX	EX
Electrolytes (mmol/l)					
Potassium	10	125	25	10	15
Sodium	105	29	120	15	100
Calcium	0.5	-	-	0.015	0.25
Magnesium	5	5	5	4	13
Chloride	10.5	20	-	32	42
Colloids (g/L)					
HES	-	50	-	-	-
PEG-35	-	-	1	-	-
Dextran 70	0.7	-	-	-	-
ROS scavengers (mmol/l)					
Allopurinol	-	1	1	-	-
Glutathione	-	3	3	-	3
Mannitol	-	-	-	30	60
Tryptophan	-	-	-	2	-
Buffers (mmol/l)					
Histidine	-	-	-	198	30
KH_2_PO_4_	-	25	25	-	-
NaHCO_3_	5	-	-	-	-
Impermeants (mmol/l)					
Citrate	30	-	-	-	-
Glucose	167	-	-	-	-
Lactobionate	-	100	100	-	80
Raffinose	-	30	30	-	-
Additives (mmol/l)					
Adenosine	-	5	5	-	-
EDTA	5	-	-	-	-
Fumarate	5	-	-	-	-
Glutamic acid	-	-	-	-	20
Ketoglutarate	-	-	-	1	-
Insulin (U/l)	-	40	-	-	-
Dexamethasone (mg/l)	-	16	-	-	-
Penicillin G (UI/l)	-	2-00	-	-	-
pH	7.4	7.4	7.4	7.20	7.3
Viscosity	Low	High	High	Low	Low
Osmolality	330	320	290	310	320-360
mOsm/kg H_2_O					

IC: intracellular, EX: extracellular.

**Table 2:** Biochemical parameters of the efficacy of storing kidneys in Biolasol and Biolasol –modified solutions with the addition of ascorbic acid and prolactin ± SD.

Time [min]	Biolasol (control group)	Biolasol +vit.C (experimental group)	Biolasol +PRL+vit.C (experimental group)	Significance
		ALT [U/l]		
2h preservation 0′	70.6±19.0	34.7±4.9	43.7±16.6	NS
2h preservation 30′	58.8±15.5	21.0±4.6	24.3±9.1	NS
48h preservation 0′	68.6±16.9	29.6±7.3	43.2±11.2	NS
48h preservation 30′	60.1±17.9	12.8±4.9	24.1±9.0	P<0.05
		AST [U/l]		
2h preservation 0′	60.5±16.4	54.3±14.0	103.4±34.6	NS
2h preservation 30′	32.6±8.9	34.9±13.6	63.9±16.8	NS
48h preservation 0′	60.3±11.1	48.0±18.7	63.8±17.5	NS
48h preservation 30′	35.8±9.4	18.0±6.0	36.0±12.3	NS
		LDH [U/l]		
2h preservation 0′	720.8±164.6	444.8±195.1	602.0±171.0	NS
2h preservation 30′	168.1±41.4	313.2±112.1	305.7±161.0	NS
48h preservation 0′	416.0±59.9	475.0±113.0	473.0±95.4	NS
48h preservation 30′	216.5±135.5	129.5±54.9	145.7±71.0	NS
		Lactates [mmol/l]		
2h preservation 0′	0.9±0.3	1.2±0.2	1.2±0.4	NS
2h preservation 30′	0.5±0.1	0.5±0.2	0.2±0.1	NS
48h preservation 0′	1.0±0.4	0.9±0.3	1.5±0.7	NS
48h preservation 30′	0.6±0.2	0.5±0.3	0.2±0.1	NS

Comparisons between the three groups were performed by analysis of variance (ANOVA) or the Kruskal-Wallis test.

## Data Availability

The data used to support the findings of this study are available from the corresponding author upon request.
